# The human squamous oesophagus has widespread capacity for clonal expansion from cells at diverse stages of differentiation

**DOI:** 10.1136/gutjnl-2013-306171

**Published:** 2014-02-26

**Authors:** Mariagnese Barbera, Massimiliano di Pietro, Elaine Walker, Charlotte Brierley, Shona MacRae, Benjamin D Simons, Phil H Jones, John Stingl, Rebecca C Fitzgerald

**Affiliations:** 1 MRC Cancer Unit, University of Cambridge Cambridge, UK; 2 Cavendish Laboratory, Department of Physics, J. J. Thomson Avenue University of Cambridge, Cambridge, UK; 3 The Wellcome Trust/Cancer Research UK Gurdon Institute, University of Cambridge, Cambridge, UK; 4 Wellcome Trust-Medical Research Council Stem Cell Institute, University of Cambridge, Cambridge, UK; 5 University of Cambridge, CRUK—Cambridge Institute, Cambridge, UK

**Keywords:** OESOPHAGEAL CANCER, EPITHELIAL DIFFERENTIATION, STEM CELLS, BARRETT'S OESOPHAGUS, EPITHELIAL PROLIFERATION

## Abstract

**Objective:**

Knowledge of the cellular mechanisms involved in homeostasis of human squamous oesophagus in the steady state and following chronic injury is limited. We aimed to better understand these mechanisms by using a functional 3D approach.

**Design:**

Proliferation, mitosis and the expression of progenitor lineage markers were assessed in normal squamous oesophagus from 10 patients by immunofluorescence on 3D epithelial whole mounts. Cells expressing differential levels of epithelial and progenitor markers were isolated using flow cytometry sorting and characterised by qPCR and IF. Their self-renewing potential was investigated by colony forming cells assays and in vitro organotypic culture models.

**Results:**

Proliferation and mitotic activity was highest in the interpapillary basal layer and decreased linearly towards the tip of the papilla (p<0.0001). The orientation of mitosis was random throughout the basal layer, and asymmetric divisions were not restricted to specific cell compartments. Cells sorted into distinct populations based on the expression of epithelial and progenitor cell markers (CD34 and EpCAM) showed no difference in self-renewal in 2D culture, either as whole populations or as single cells. In 3D organotypic cultures, all cell subtypes were able to recapitulate the architecture of the tissue of origin and the main factor determining the success of the 3D culture was the number of cells plated, rather than the cell type.

**Conclusions:**

Oesophageal epithelial cells demonstrate remarkable plasticity for self-renewal. This situation could be viewed as an ex vivo wounding response and is compatible with recent findings in murine models.

**Significance of this study**

**What is already known on this subject?**

- The human oesophagus is a multistratified squamous epithelium, in which cell proliferation is restricted to the basal and the first few suprabasal layers.
- Stem cells are responsible for tissue maintenance in the GI tract; however, clear delineation of stem cells in the oesophagus is still lacking.
- Conflicting results have been generated on this topic using 2D models; hence, a 3D approach is needed to elucidate the functional architecture of this tissue.

**What are the new findings?**

- The most quiescent cells expressing putative stem cell markers are located at the tip of the papillae.
- Asymmetric division, which is a hallmark of stem cells, is not restricted to a specific cell compartment.
- Cells at diverse stages of differentiation sorted according to progenitor cell markers have equal capacity for self-renewal and ability to reconstitute a squamous 3D architecture in vitro.

**How might it impact on clinical practice in the foreseeable future?**

- In the oesophagus, the ability for tissue repair and renewal is not dependent on cells with stem cell-like properties. These findings may be important for our future understanding and exploitation of the oesophageal response to injury such as inflammation and carcinogenesis.

## Introduction

The human oesophageal stratified squamous epithelium is maintained through an exquisite balance between proliferation and terminal differentiation.^1^ Most of the current knowledge on tissue homeostasis and injury repair is based on murine models; however, there are fundamental differences between mouse and human oesophagus. First, the human oesophagus is non-keratinising, hence more vulnerable to abrasive, thermal and pH injuries. Second, the posture of humans creates a different anatomical relationship between the oesophagus, diaphragm and stomach, which normally functions to protect from gastro-oesophageal reflux. When this antireflux barrier is disrupted, the chronic exposure of the oesophagus to acid and bile can lead to inflammation and precancerous metaplasia called Barrett's oesophagus.^2^ Furthermore, murine oesophagus tissue architecture is simpler than in humans since it lacks crypts and gland structures.^3^

In squamous epithelia, proliferation is generally confined to the basal layer. On commitment to terminal differentiation, basal cells exit the cell cycle and migrate towards the luminal surface from which they are shed. The prevailing dogma has been that a discrete population of long-lived stem cells is responsible for tissue maintenance.^1^ Hence, identification of stem cells or functionally equivalent cells is paramount to unravel the mechanisms involved in carcinogenesis.^4^ A paradigmatic example is the identification of LGR5+ cells in the intestinal epithelium and the demonstration of their role in self-renewal and colonic tumourigenesis.^5^ ^6^ In the mouse oesophagus, various methods have been used to track stem cells. α6-intergrin and the transferrin receptor (CD71) were used to track a discrete population of cells with a slightly longer cell cycle, but no difference was found between these cells and other basal cells in terms of colony forming ability, suggesting that they were not functionally distinct.^7^ Using Hoechst dye extrusion, an oesophageal subpopulation was identified with the ability to self-renew and give rise to differentiated suprabasal cells in a 3D organotypic culture.^8^ More recently, with a transgenic label-retaining assay approach coupled with 3D imaging, Doupe and coworkers failed to identify quiescent epithelial stem cells in the murine oesophagus and found that progenitor cells contribute equally to wound repair.^9^ This finding might explain the heterogeneous response of oesophageal cells in culture since this environment resembles wounding and may intrinsically alter cell behaviour.^10^

In humans, the multiple layers of proliferating cells and the irregular papillary and glandular structures make interpretation of proliferative cell behaviour more complex than in the mouse, especially when relying on conventional 2D sections. Initially, based on staining of paraffin sections for proliferating cell nuclear antigen, it was suggested that quiescent, putative stem cells were located at the top of the papillae.^11^ Subsequently, a study employing fluorescent cell sorting methodologies found that the interpapillary basal layer was relatively quiescent and had a high proportion of asymmetrical mitoses, both characteristics suggestive of stem cells.^12^ More recently, a lineage-tracing experiment was performed using 5-iodo-2′-deoxyuridine in four patients undergoing oesophagectomy.^13^ Label-retaining cells were found in the papillae of the oesophageal squamous basal layer, and investigation of metaplastic Barrett's and gastric tissues performed in the same patients revealed that all of these tissues had small populations of slow-cycling, uncommitted cells in discrete locations suggestive of a stem cell niche.^13^ None of these human studies examined the self-renewing capacity of these putative stem cell populations in comparison to other cell compartments using 3D in vitro and in vivo models or tested the hypothesis raised by the mouse data that all cycling cells are equivalent.

The aims of this study were therefore to use 3D imaging, coupled with staining for a range of cell lineage markers, to investigate the patterns of proliferation and mitosis in the human oesophageal epithelium. We also sought to determine the ability of distinct subpopulations of cells to self-renew using in vitro and in vivo assays.

## Methods

### Human tissue sample collection

Tissue samples from normal oesophagus were obtained following research ethics approval and individual informed consent from patients who underwent oesophagectomy for oesophageal cancer (REC# 07/H305/52) or from cadaveric patients whose next of kin have consented to the donation of their relative's oesophagus for research purposes (REC# 11/EE/0253). Macroscopically normal squamous mucosa adjacent to the proximal resection margin was sampled from cancer resection specimens, whereas a 10 cm segment of whole oesophagus was retrieved from organ donor patients with a normal oesophagus.

### Cell lines

NIH 3T3 (ACCT) and NAF1^14^ were cultured at 37°C, 5% CO_2_ in Dulbecco's Modified Eagle's Medium (DMEM) supplemented with 10% foetal calf serum (PAA) to provide feeder layer for the culture in 2D and 3D, respectively, of primary oesophageal cells.

### Immunofluorescence (IF) and confocal imaging on epithelial whole mounts

Normal oesophageal samples were collected in 5 mM EDTA phosphate buffered saline (PBS), then processed and stained as described in online supplementary methods.

Images were captured using an upright Zeiss LSM 510 META Confocal Microscope and the LSM 510 Software V.3.2. z-stacks or sequential images were processed with Volocity©4.2 (2007, Improvision Ltd. Software) to obtain 3D reconstructions and snapshots.

**Figure 1:**
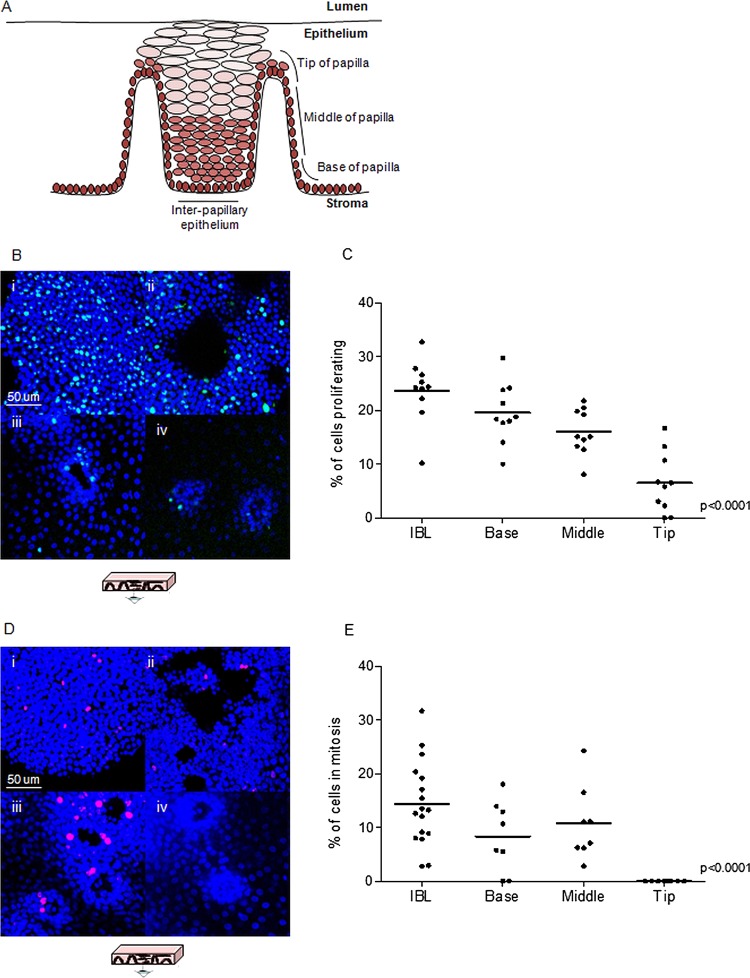
Compartmentalisation and quantitative analysis of proliferation and mitosis. (A) Representation of a section of human oesophageal epithelium. (B) Whole mounts stained for the proliferation marker Ki67 (green) representative of interpapillary basal layer (IBL, i), base (ii), middle (iii) and tip (iv) of the papillae. (C–E) Quantitative analysis of proliferation (C) and mitosis (E) in the four-cell compartments expressed as percentage of Ki67 and PH3-positive cells, respectively. Data points represent the average proliferation rate in each individual whole mount or patient sample and the statistical analysis assesses the significance of the overall trend across the four regions. (D). Whole mounts stained for the mitotic marker PH3 (red) representative of interpapillary epithelium (i), base (ii), middle (iii) and tip (iv) of the papillae. Blue colour identifies DAPI nuclear staining.

For the quantitative analysis, z-stacks of the interpapillary epithelium (up to the fifth layer of cells) or sequential z-stacks reproducing the whole thickness of the papillae (up to three layers) were acquired from samples stained for Ki67 or phospho-histone PH3. All the cells were counted (∼4×10^4^ in total) and the percentage of Ki67+ or PH3+ cells (representing proliferating and mitotic rates, respectively) was calculated for four specific compartments of the epithelium: the interpapillary epithelium, the bottom, the middle and the tip of the papillae (figure 1A). The methodology used to measure the orientation of mitoses is described in online supplementary methods.

### Flow cytometry

Squamous oesophageal samples were collected in DMEM, washed in PBS and incubated with 500 μg/mL dispase (Sigma) at 37°C for 30 min. The epithelial sheet was peeled off, minced and incubated at 37°C for 45 min in 0.25% Trypsin EDTA (Invitrogen). The cell suspension was then passed through a 70  μM cell strainer, spun and washed in PBS.

The cells were incubated with 4',6-diamidino-2-phenylindole (DAPI) (Roche) and the antibodies of interest for 10 min on ice, washed and resuspended in PBS. The primary antibodies that were used are listed in online supplementary table S1. Biotin-conjugated antibodies were detected with strepavidin-APC-Cy7 (Biolegend). Fluorescence-activated cell sorting was carried out according to the level of binding of the antibodies. Sorted cells were selected through sequential gating (see online supplementary figure S3A) based on forward scatter (FSC), side scatter (SSC), trigger pulse width, to gate out doublets, DAPI to gate out dead cells and CD31/CD45 binding to exclude haematopoietic and endothelial cells. Cells were gated and sorted based on the expression of CD34 and EpCAM. FITC-conjugated IgG1 raised in mouse (Dako) was used as an isotype control. The ABC Anti-Mouse Bead Kit (Invitrogen) was used for adjusting compensation. Data were analysed using FlowJo software (Tree Star, Inc, Ashland, Oregon, USA).

### q-PCR characterisation of sorted cells

Reverse transcription (RT) and cDNA amplification were carried out in one step using the SuperScript III One-Step RT-PCR System (Invitrogen). Prior to RT, a primer mix was prepared containing 200 nM of forward and reverse primers of each individual gene of interest and three housekeeping genes (GAPDH, β-actin and RPS18). Briefly, for each reaction 100 flow-sorted cells were incubated with 0.2 µL SuperScript III RT Platinum Taq Mix and 2.5 µL of primer mix in a final volume of 9 µL. Incubation conditions were as follows: 15 min at 50°C for RT, 2 min at 95°C for Taq inactivation, followed by a touch-down PCR amplification with 20 cycles of 15 s at 95°C for denaturation and 4 min for annealing and extension (decreasing temperature from 70°C down to 60°C over 5 cycles, followed by 15 cycles at 60°C) . Amplification was followed by DNA digestion with Exonuclease 1 (Exostar-1 step, GE Healthcare) as per the manufacturer's recommendation. qPCR was performed with LightCycler 480 SYBR Green I master (Roche Diagnostics Ltd, Rotkreuz, Switzerland) and 0.2 µM forward and reverse primer in a final volume of 10 µL using LightCycler 480 technology (Roche). qPCR consisted of 55 cycles of denaturation at 95°C (10 s), annealing at 60°C (15 s) and extension at 72°C (15 s). Primer sequences are provided in online supplementary table S2. Results were analysed with LightCycler 480 software V.1.5 (Roche). The cycle threshold Cp was automatically calculated in second derivative maximum method, and the expression of each gene relative to three housekeeping genes was calculated as ΔCp. Experiments were done in triplicate.

### Immunofluorescent characterisation of sorted cells

Sorted cells were collected in PBS and spun onto positively charged glass slides using a Cytospin2 centrifuge (Thermo-Scientific) and fixed for 5 min with 4% paraformaldehyde (PFA). Cells were then incubated with a permeabilization/blocking buffer (0.25% fish skin gelatine, 0.2% Triton X-100, in PBS) for 15 min, primary antibody (PanCK—pancytokeratin) (see online supplementary table S1) for 1 h at RT, washed three times in PBS and incubated for 1 h at RT with AF647-goat antirabbit (Invitrogen). The slides were mounted with DAPI Vectshield (Vector Laboratories). Images were captured using an upright Zeiss LSM 510 META Confocal Microscope and the LSM 510 software.

### Colony forming cell assay and characterisation of epithelial lineage of cell clones

NIH 3T3 mouse fibroblasts were inactivated using mitomycin, plated on 60 mm dishes (1.5×10^6^ cells/dish) and incubated at 37°C, 5% CO_2_. After 24 h, oesophageal cells were sorted into 5% fetal bovine serum (FBS) DMEM in 2 mL Eppendorf tubes, plated in the dishes containing the inactivated fibroblasts and cultured in FAD medium (composition in online supplementary methods). After 2 weeks, the number of clones generated in each dish was counted and the lower limit of clone size scored was 22 cells. Full images of the clones after 2 weeks of culture were acquired and the cells in each clone were counted manually using the NIH Image J software.

Immunofluorescence staining for proliferation and cell lineage markers on representative clones was performed as above (in online supplementary table S1). Secondary antibodies used were FITC-goat antimouse (Vector Laboratories) and AlexaFluor-647-goat antirabbit (Invitrogen). Images were captured with an inverted Zeiss Axio Observer D.1 microscope and AxioVision software.

### Single cell sorting

NIH 3T3 mouse fibroblasts were inactivated as above and seeded in 96-well plates (2×10^4^/well). After 24 h, DMEM was replaced with FAD medium and oesophageal cells were sorted individually in each well. Sufficient cells to fill six plates for each of the four populations gated were normally sorted. Cells were then incubated at 37°C, 5% CO_2_ and the number of wells containing clones was counted after 2 weeks. IF was performed as above on representative clones.

### 3D organotypic culture

NAF1 cells were embedded in collagen and rafts were prepared as described previously.^15^ After 24 h, oesophageal cells from primary cultures were sorted in 5% FBS DMEM in 2 mL Eppendorf tubes, resuspended in FAD medium, plated on the collagen rafts and incubated at 37°C, 5% CO_2_. The number of cells seeded in each raft varied from 2000 to 200 000. After 4 days, the rafts were lifted onto a mesh and cultured at an air–liquid interface for 14–21 days. The rafts were then fixed in 4% PFA and embedded in paraffin blocks.

### Histology and immunohistochemistry

Paraffin-embedded blocks of in vitro-derived outgrowths were cut at a thickness of 7 μm and the sections were placed on polylysine slides. These sections were then immunostained to detect the expression of a panel of proliferation and lineage markers (see online supplementary table S1) using the BOND MAX automated system (Leica) according to the manufacturer's instructions. Images were acquired with a BX41 upright brightfield microscope (Olympus).

### Statistical analysis

A Jonckheere–Terpstra test was carried out using SPSS V.16.0 to analyse the trend of the proliferation and mitotic rates across the four-cell compartments. Graphpad Prism (V.5) was used to perform a non-parametric Kruskal–Wallis test for the statistical analysis of the clonogenic potential of the sorted cell populations. Results were considered statistically significant when p<0.05.

## Results

### Quantitative analysis of proliferation and mitosis in the human squamous oesophagus

Proliferation (three areas for each of n=10 different patients) and mitosis (three areas for each of n=8 patients) were quantified in whole mounts. The tissue was divided into four-cell compartments comprising the interpapillary epithelium, the base, middle and tip of the papillae (figure 1A). Cycling cells were mainly located in the basal layers extending to the 5th–6th suprabasal cell layer (figure 1B). There was a continual decrease in the proliferation rate from the interpapillary epithelium towards the top of the papilla (figure 1C). The staining for PH3 showed a similar decreasing trend in the mitosis rate (figure 1E). At the tip of the papilla, there was no evidence of cell division; specifically no mitotic figures were detectable at this location in any of the samples (figure 1D).

Previous data suggest an asymmetric plane of division for stem cells, in which one cell is retained in the basal layer and the other migrates luminally and differentiates. We measured the orientation of mitoses with respect to the plane of the basal layer (see online supplementary figure S1A,B), and in the three-cell compartments where mitotic figures were identified the orientation appeared randomly distributed (see online supplementary figure 1C).

### Lineage markers expressed in quiescent cells at the papillary tips

**Figure 2:**
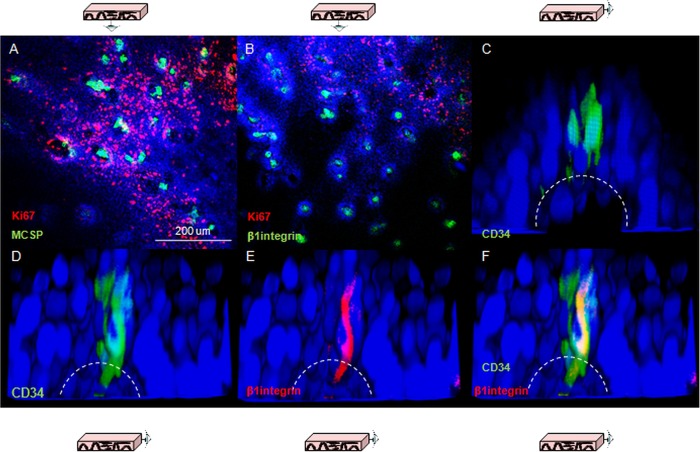
Putative epithelial stem cell markers in human oesophageal whole mounts. (A, B) Human oesophageal epithelium following labelling for melanoma chondroitin sulfate proteoglycans (green, A) and β1-integrin (green, B); shown in red is Ki67 labelling. (C) 3D projection of a confocal z-stack of the tip of papillae in following labelling for CD34 (green). The dashed lines represent the border of the basal layer. (D–F) 3D projection of a confocal z-stack of the tip of a papilla following labelling for CD34 (green, D) and β1-integrin (red, E). (F) z-stack with the merged staining. DAPI was used as counterstain (blue).

Three putative epithelial stem cell markers were used to investigate whether the gradient of proliferation observed along the axis of the papilla reflect different cell lineages. β1-integrin has been characterised in human skin,^16^ CD34 has been proposed as an epithelial stem cell marker in mouse oesophagus^8^ and melanoma chondroitin sulfate proteoglycans (MCSP) has been identified as a potential marker for cells expressing high levels of β1-integrin.^17^ Consistent labelling for all of these markers was obtained in quiescent cells located at the top of the papillae (figure 2A–C). Co-staining for β1-integrin and CD34 clarified that the population of CD34 cells also express β1-integrin (figure 2D–F).

**Figure 3:**
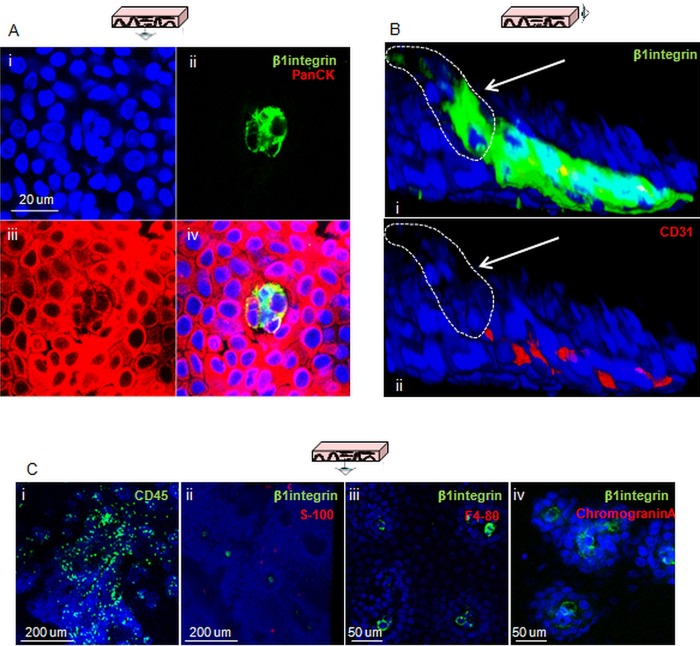
Confirmation of the epithelial lineage of cells expressing putative stem cell markers and exclusion of other possible cell origins. (A) Confocal section of the tip of a papilla following labelling for β1-integrin (green) and PanCK (red). (B) 3D projection of a confocal z-stack of the tip of a papilla following co-staining for β1-integrin (i, green) and the endothelial marker CD31 (i and ii, red); the white arrows in both pictures indicate the cells that express β1-integrin but not CD31. (C) Confocal sections of oesophageal whole mounts acquired following staining for the lymphocyte marker CD45 (i, green), β1-integrin and the neural crest marker S-100 (ii, green and red, respectively), β1-integrin and the macrophage marker F4-80 (iii, green and red, respectively), β1-integrin and the Merkel cell marker chromogranin A (iv, green and red, respectively). DAPI was used as counterstain (blue).

Cells expressing β1-integrin also expressed the epithelial marker PanCK (figure 3A). Immune labelling of markers for other cell lineages was also used to exclude the possibility of non-epithelial origins for these cells. Negative labelling for any given cell lineage marker compared with controls, a lack of co-labelling with β1-integrin or labelling with a completely different pattern compared with that of β1-integrin were considered sufficient evidence to discount a given cell lineage. Double labelling for β1-integrin and the endothelial marker CD31 highlighted a distinct population of β1-integrin-positive and CD31-negative cells at the top of the papilla, suggesting that they are not endothelial in origin (figure 3B). Endothelial cells located inside the lumen of the papilla were both CD31 and β1-integrin-positive. The pan leucocyte marker CD45 and the melanocyte S-100 showed a lack of co-staining with β1-integrin, and a different expression pattern from that observed for β1-integrin (figure 3C). Co-staining for β1-integrin and the macrophage marker F4-80 showed that the latter was not expressed in these samples (figure 3B); the validation of the antibody is shown in online supplementary figure S2. A similar result was obtained for the Merkel cells marker Chromogranin A, which excluded a neuroendocrine origin for β1-integrin-positive cells (figure 3B, see online supplementary figure S2). These results suggest that the quiescent β1-integrin/CD34-positive cells at the tip of the papillae are epithelial and part of the basal layer.

### Clonogenic potential of phenotypically distinct oesophageal subpopulations

**Figure 4:**
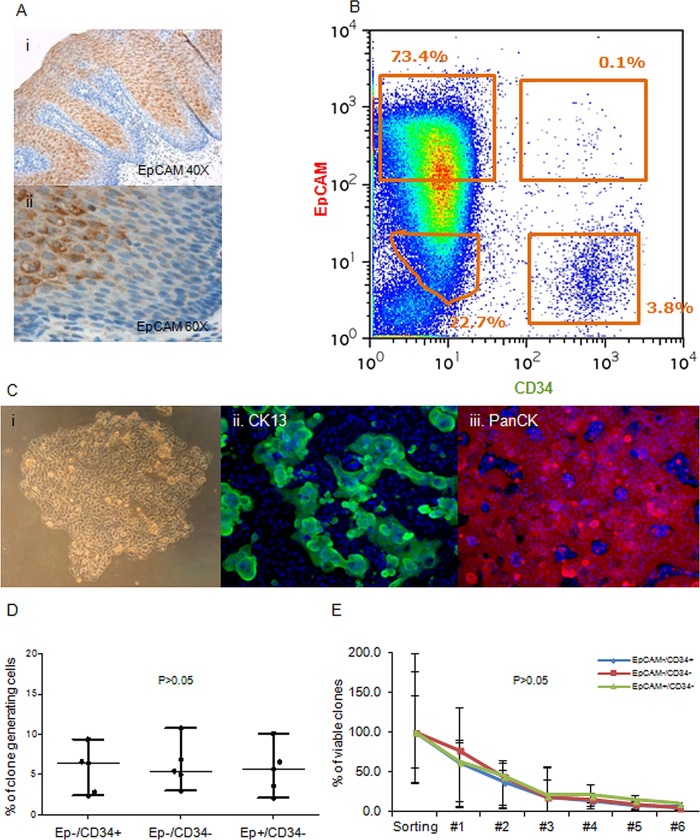
Growth properties of phenotypically distinct subsets of human oesophageal cells. (A) Immunohistochemistry staining for EpCAM in normal human oesophagus ×20 (i) and ×40 (ii). (B) Representative flow cytometry dot plot used to sort oesophageal epithelial cells based on the expression of EpCAM and CD34. (C) Example of a colony generated by a CD34+EpCAM− cell after 14 days in culture (i) and immunostaining for CK13 (ii) and PanCK (iii) DAPI was used as counterstain (blue). (D) Cloning efficiencies of the three-cell populations seeded as single cells in 96-well plates. (E) Self-renewal of the clones obtained following single cells sorting for CD34 and/or EpCAM expressed as percentage cells of the clones initially generated from the sorting experiment that remained viable after each passage.

In order to investigate the self-renewal potential of CD34-positive (CD34+) cells in comparison to the other cell populations, we performed cell sorting on samples of squamous epithelium. CD34 was selected in preference to β1-integrin or MCSP since this antibody was the most reliable and gave the most reproducible results (data not shown). To distinguish between epithelial cells in the suprabasal layer from less differentiated basal cells, CD34 was combined with EpCAM, an epithelial marker that is broadly expressed in suprabasal cells (figure 4A). The sorting showed that the majority of cells were CD34-negative (CD34−), with a gradient of expression of EpCAM with a smaller fraction of CD34+ cells. For the functional analysis of different cell populations, we selected cells with highest level of EpCAM (EpCAM+) and lowest level of EpCAM (EpCAM−) and sorted three distinct cell types (CD34−/EpCAM−, CD34+/EpCAM−, CD34−/EpCAM+) and a fourth smaller and less discrete group of cells that were CD34+/EpCAM+ (figure 4B). Validation was carried out using IF and qPCR to confirm the cell lineage of the sorted subpopulations. The four cell groups contain a high proportion of epithelial cells that are positive for the epithelial marker PanCK (see online supplementary figure S3B). By qPCR we found that the double-negative population showed predominant epithelial/squamous features with relatively low vimentin expression, indicating a low level of cell contamination from the stroma. CD34−/EpCAM+ cells have a clear epithelial, squamous profile. CD34+/EpCAM− cells showed expression of squamous epithelial markers, but also very high levels of expression of mesenchymal and immune-related markers, indicating a higher stromal content (see online supplementary figure S3C). The cells sorted as double-positive show a profile very similar to the CD34+/EpCAM− cells; however, the poorly defined position of these cells in the sorting dot-plot (figure 4B) and the extremely small size of the cell fraction itself suggest that this group of cells could be an artefact constituted mostly of doublet cells with high CD34 levels and medium/low EpCAM levels. For this reason, the CD34+/EpCAM+ cells were not used in the subsequent experiments.

Following cell sorting, the three populations were plated into a colony forming cell (CFC) assay and clone-size analysis was carried out on 74 of these clones 7 days after plating. No difference in clone size was observed and the clones underwent senescence after seven passages. IF for specific lineage markers that the clones obtained were composed of epithelial squamous cells (figure 4C). In order to ensure that subtle differences were detected, a quantitative analysis of the number of clones generated was carried out on at least seven experimental repeats per cell population, which showed that all four cell populations have the same or a comparable clonogenic potential (see online supplementary figure S3D). To minimise the effect of a large variation in the number of cells plated due to the different size of these cell populations at extraction (figure 4B), we next performed single-cell sorting into 96-well plates, which showed that the three main subpopulations of cells have a very similar cloning efficiency in each experiment (figure 4D). These clones were then ‘passaged’ several times to determine self-renewal capacity of individual cell fraction, but no difference was observed in the number of viable clones with increasing passage (figure 4E).

### Self-renewing potential of oesophageal cells in organotypic in vitro 3D models

**Figure 5:**
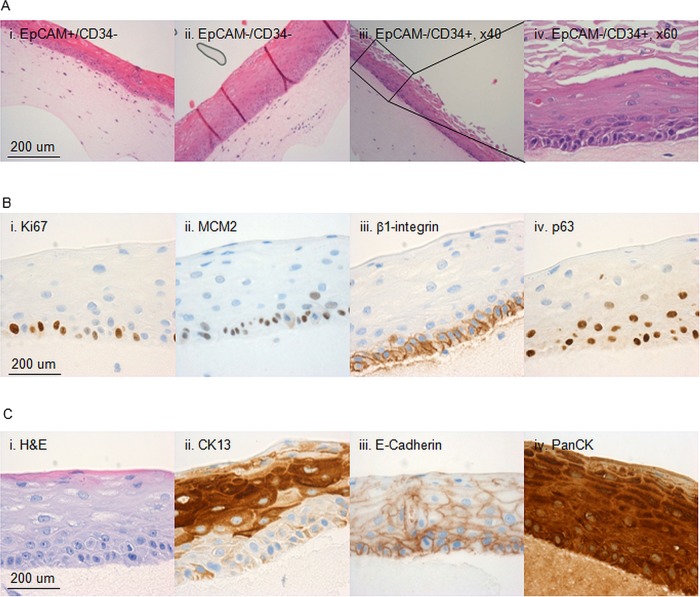
Characterisation of the 3D in vitro cultures. Characterisation of 3D organotypic cultures from oesophageal cells sorted on the basis of EpCAM and CD34. (A) Representative images of 3D organotypic cultures grown stained with haematoxylin and eosin (H&E) from CD34−/EpCAM+ cells (i), CD34−/EpCAM− cells (ii) and CD34+/EpCAM− cells (×20 M, ×40, iii and iv, respectively). (B) Representative images of outgrowths stained for Ki67 (ii), MCM2 (ii), β1-integrin (iii) and p63 (iv). (A) Representative images of the same outgrowths in H&E (i), or stained for CK13 (ii), E-Cadherin (ii) and PanCK (iv).

We used 3D organotypic in vitro culture system to assess the ability of the isolated cell subfractions to regenerate structures recapitulating the tissue of origin. All the cultures that were seeded with a minimum of 2000 sorted cells, irrespective of cell phenotype, generated multistratified outgrowths with morphological features of differentiated squamous epithelium (figure 5A).

Next we performed i analysis on the 3D organotypic cultures with a panel of cell lineage, differentiation and progenitor markers to compare the features of the regenerated tissue with the native oesophagus. The progenitor marker for a stratified epithelium p63 was expressed in the basal layer as well as β1-integrin (figure 5B). Pan-epithelial markers (PanCK and E-Cadherin) were expressed throughout the stratified structure (figure 5C) and specific marker of squamous differentiation (CK13) confirmed the correct cell lineage (figure 5C). The multistratified epithelia also maintained characteristics of self-renewing and proliferating tissue since cells in the basal layer express proliferation markers Ki67 and MCM2 (figure 5B).

## Discussion

Using a combination of 2D and 3D ex vivo and in vitro models, we have carried out a descriptive and functional analysis of the proliferation, cell division and self-renewal in the human oesophagus. We found a decreasing gradient of proliferation from the interpapillary basal layer to the tip of the papillae, which appears to contain a niche of CD34 quiescent cells. However, clonogenic potential and the ability to reconstitute a 3D stratified epithelium were not restricted to the CD34 population.

Our observations contrast with previous studies that showed that the interpapillary basal layer is the cell compartment with the lowest rates of proliferation and mitoses, highest rate of asymmetrical mitoses,^12^ ^18^ but are in agreement with an earlier study that found the lowest proliferation rate at the tip of the papillae.^11^ An explanation to this could be that previous studies relied on 2D sections, which can expose to artefacts due to cross-cutting and branching papillae.^19^

We also observed that the quiescent cells at the tip of the papilla expressed putative stem cell markers (β1-integrin, MCSP and CD34). β1-integrin is the best characterised progenitor marker in squamous epithelia.^16^ ^20^ In our in vitro 3D oesophageal culture, β1-integrin was consistently expressed throughout the basal layer, suggesting that the entire basal layer is in an undifferentiated state. MCSP has been proposed as a marker of a subpopulation of cells highly expressing β1-integrin,^17^ and our results are in keeping with this finding. CD34 is a well-established haematopoietic stem cell marker and was proposed as stem cell/progenitor marker in mouse oesophagus.^8^

Surprisingly, CD34+/EpCAM− cells from the basal layer did not have greater clonogenic potential than other cell fractions and all sorted populations had similar growth and differentiation properties. One explanation would be that, in culture, the cells are no longer in a homeostatic situation, but are in a non-physiological condition. Injury models have been better characterised in mouse using wounding methods^8^ or endoscopic biopsy and lineage tracing.^9^ In the first study, after injection of CD34 cells into a site of injury, only CD34 cells participated in the reconstitution of the tissue. In the second study, genetic lineage tracing, transgenic cell proliferation and 5-ethynyl-2′-deoxyuridine (EdU) demonstrated that all proliferative cells contribute equally to repair in vivo a wound postendoscopic biopsy. The same study found evidence in support of one cell population switching in response to damage between balanced stochastic fate and cell proliferation. The results of this study are consistent with this theory and suggest that quiescent cells at the top of the papillae would not have a privileged role in these processes.

Lineage-tracing models are the ultimate strategy to prove the existence of stem cells as progenitors but can generally only be applied to animal models. Pan *et al*^13^ were recently able to track label-retaining cells in human oesophagus 29 days after injecting the patients with 5-iodo-2′-deoxyuridine. However, this model allows for histological rather than functional investigation as only a single time point can be analysed and the cells cannot be traced for a specific molecular marker. Further investigations using systems such as viral vectors in 3D in vitro models could elucidate the molecular characteristics of cells that maintain the human oesophageal epithelium.

The evidence from this paper and the recent findings in mouse oesophagus suggest that progenitor cells, which can respond to injury and regenerate tissue, are widespread and are not restricted to the basal layer and include cells that have already committed to epithelial differentiation. It is also important to note that the present work focused completely on the assessment of cell behaviour in the epithelium and did not evaluate the contribution of oesophageal submucosal glands that have also been shown to be a potential source of cells with the capacity to self-renew and alter their fate in metaplasia.^21^ ^22^

We have also applied an in vitro model to sorted cells and optimised it for the use of a smaller number of cells per sample compared with previous models. This could be used to investigate the molecular factors involved in the conversion of squamous epithelium to Barrett's oesophagus. Transgenic mouse models have been recently used to investigate the possible cellular origin of Barrett's oesophagus.^23^ ^24^ However, the significant difference in the oesophageal anatomy between human and mouse precludes the full translation of this knowledge into the human kind, which still remains an active area of debate.

In conclusion, this study confirms the existence of a quiescent cell population at the tip of the papillae and goes against the idea that asymmetric division occurs more frequently in slowly cycling cells in the basal layer. In keeping with recent data in mouse, we suggest that in vitro clonogenic potential is not confined to a distinct cell population. This knowledge will contribute to our growing understanding of homeostasis in human oesophagus and will lead to a better understanding of carcinogenesis through application of human model systems described herein.

## Supplementary Material

Web supplement

Web figures

Web table 1

Web table 2
