# Supplementary material for: The human squamous oesophagus has widespread capacity for clonal expansion from cells at diverse stages of differentiation
Source: Gut. 2014 Feb 26;64(1):11–9. doi: 10.1136/gutjnl-2013-306171 (PMC4283695; doi:10.1136/gutjnl-2013-306171)
Supplement: Web supplement [file gutjnl-2013-306171-s1.pdf]

SUPPLEMENTARY INFORMATION

Supplememantary Methods

Processing and staining of epithelial wholemounts. The fresh oesophageal samples collected in 5mMPBS were washed with PBS and incubated in 5mM EDTA PBS for 3 hrs at room temperature (RT). The intact epithelial sheet was peeled from the stroma, fixed with 4% paraformaldehyde (Sigma) for 2 hrs at RT, washed with PBS and then stored at 4 °C in 0.2% sodium azide PBS (Sigma).

Small fragments (2-3 mm^2) of fixed squamous oesophageal epithelium or whole fixed Barrett's biopsies were incubated for 30 mins in PB buffer (0.5% Skimmed milk powder, 0.25% fish skin gelatine, 0.5% Triton X-100, in Tris-buffered saline). Whole-mounts were then incubated overnight with primary antibody (Supplementary Table 1) at room temperature and washed in Tris-buffered saline/Tween (TBS/T, 0.2% Tween 20 in TBS) for 4 hrs. Incubation with the secondary antibody (FITC anti-mouse, Vector Laboratories; Alexafluor®546 anti-mouse, anti-rabbit or anti-rat, Alexafluor®647 anti-mouse, Invitrogen) diluted in PB buffer was performed as above. Double staining was performed with primary antibodies raised in different species. In two cases (co-staining for β1-integrin and CD31 or CD34) this was not possible; and therefore the double staining was performed by staining the samples initially for CD31 or CD34 and then re-fixing the tissue as above and incubating it with the FITC-conjugated antibody for β1-integrin, which therefore did not require a secondary antibody staining that would have reacted with the primary antibody used in the first staining. All samples were washed before being mounted on a slide with Vectashield™ DAPI (Vector Laboratories). Most of the antibodies used for the validation of the epithelial lineage of β1-integrin+ cells were raised in the same species (mouse) as the β1-integrin+ antibody. For this reason we proceeded to a first staining with each lineage marker alone and carry out a double staining only if the expression pattern of the marker was the same as the one of β1-integrin+. In the specific case of the immune marker CD45, a single staining was deemed sufficient to show that β1-integrin antibody

does not stain CD45+ cells, because the distribution of CD45+ cells was very different from the distribution of β1-integrin+ cells.

Measurement of mitosis orientation. A total of 120 z-stacks (38 in the inter-papillary epithelium, 43 at base of papillae and 39 in the middle of the papillae) of nuclei expressing the mitotic marker PH3 were acquired. From their 3D projections, the angle between the axis of the mitotic spindle and the plane of the basal layer was measured manually (Supplementary Figure 1). The results are expressed as a distribution of mitotic couples in intervals of angle amplitude and the data were appropriately normalized to account for the space volume as described in Supplementary Figure 1.

Composition of FAD Medium. DMEM high Glucose/Hams F12 1:1, supplemented with 5% fetal bovine serum, 1.8x10-4M adenine (Sigma-Aldrich), 0.5ug/ml hydrocortisone (Fisher Scientific), 10^{-10M} cholera toxin Enzo LifeScience), 10ng/ml epidermal growth factor (peprotech), 1:1000 Gentamicin/Amphotericin B (Invitrogen) and 5ug/ml insulin (Sigma Aldrich)).

Supplementary legends

Supplementary Table 1. Primary antibodies. IHC = Immuno-histochemistry, IF = Immuno-fluorescence, FC = Flow cytometry, FITC = Fluorescein isothiocyanate, PE = Phycoerythrin.

Supplementary Table 2. Primers used in amplification and qPCR.

Supplementary Figure 1. Quantitative analysis of the orientation of cell division. (*A***)** Representation of the measurement of the angle described by the plane of the basal layer and the axis of mitosis. Once the measurements were acquired, an appropriate normalisation was necessary to account for bias due to the metric, i.e. even if the axis of cell division were chosen randomly, dividing cells with a mitotic orientation parallel to the plane of the basal layer would be found in higher abundance than those that are perpendicular, simply due to the larger phase space of possibilities. This is represented by the areas filled with the stripe pattern that become gradually

smaller with the increases of the angle of cell division. This bias can be removed by re-normalizing the distribution by the factor *A/Cos(θ)*, where θ denotes the angle measured with respect to the basal layer and *A* denotes a constant normalization factor. (*B*) Examples of z-stack projections used for the measurement of the angle of cell division, the red staining (PH3) identifies the mitotic figures. i and ii represent typical figures in the inter-papillary basal layer and in the middle of the papillae, respectively. Since the base of the papillae is a transition compartment between the other two, mitotic figures have been assessed based on either of the two examples in panel *C* depending on the position of the cells, that is closer to the neck of the papillae or to the inter-papillary epithelium. (*C*) Normalised percentage distribution of mitotic figures based on the amplitude of the angle of cell division in the inter-papillary basal layer (n=39), the basal layer at the base (n=44) and in the middle (n=40) of the papillae.

Supplementary Figure 2. Validation of antibodies used for characterization of cell lineage and that were not expressed in human oesophageal wholemount. (A-B). The macrophages marker F4-80, was validated in FFPE blocks of blood cells and stained using Immunohistochemistry (A) and Immunofluorescence (B) techniques. C-D. The Merkel cells marker Chromogranin A was expressed in HeLa cells using both Immunofluorescence (C) and Immunohistochemistry (D) techniques.

Supplementary Figure 3. Flow-sorting gating strategy and characterisation of sorted human oesophageal cells. (A) Colour-coded flow-cytometry plots that describe the sequential gating strategy for cell sorting. Firstly cells were gated based on morphology and size to exclude cell debris (i), the cells were then further gated sequentially to eliminate doublets (ii), to exclude dead cells that bind DAPI (iii) and to gate out part of the endothelial/immune cells that bind CD31 and CD45, respectively (iv). The population gated in the last plot (iv) represents the cells actually sorted. (B) Immuno fluorescence labelling of freshly sorted cells for the pan epithelial marker Pan Cytokeratin. (C) qPCR analysis of the 4 cell fractions sorted for p63, CK13, β1-integrin and vimentin. (D) Quantitative analysis of the clonogenic potential of the cell populations after sorting for CD34 and EpCAM and cultured as whole cell population expressed as percentage of cells generating clones. It

should be noted that the scale of the clonal efficiency in this panel cannot be compared directly with Fig 4D due to methodological differences in the FACS and culture protocols for the single-cell and sub-population experiments.
