# Supplementary figures and images for: The human squamous oesophagus has widespread capacity for clonal expansion from cells at diverse stages of differentiation
Source: Gut. 2014 Feb 26;64(1):11–9. doi: 10.1136/gutjnl-2013-306171 (PMC4283695; doi:10.1136/gutjnl-2013-306171)

Supplementary Figure 1

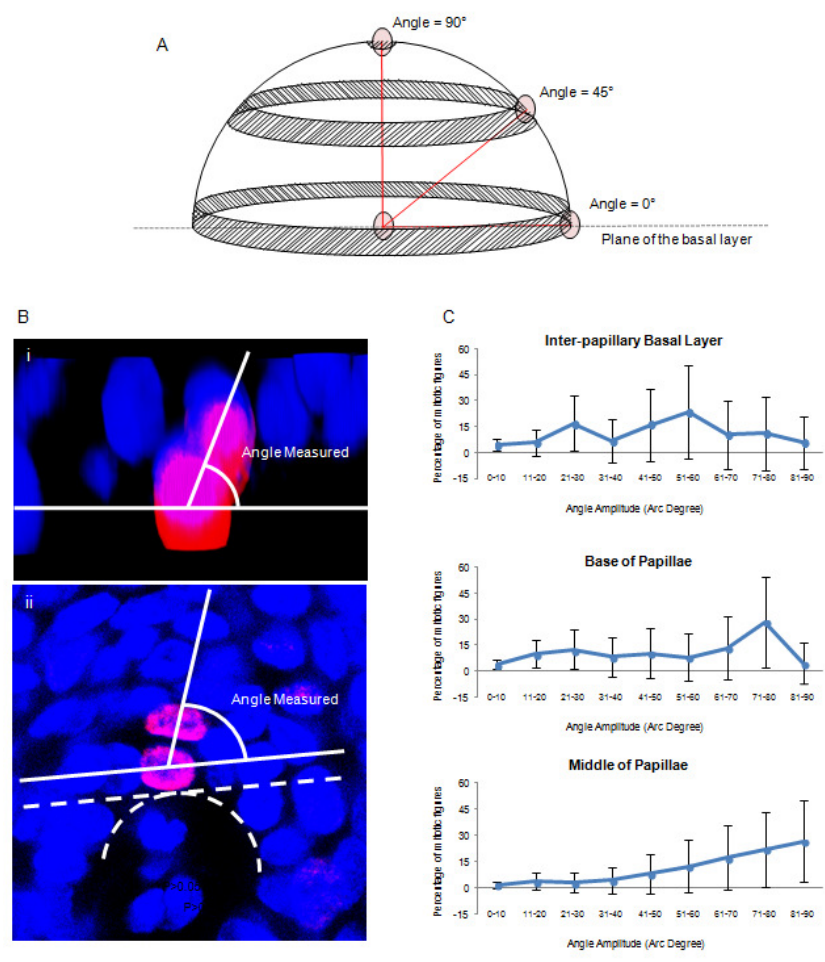

Supplementary Figure 2

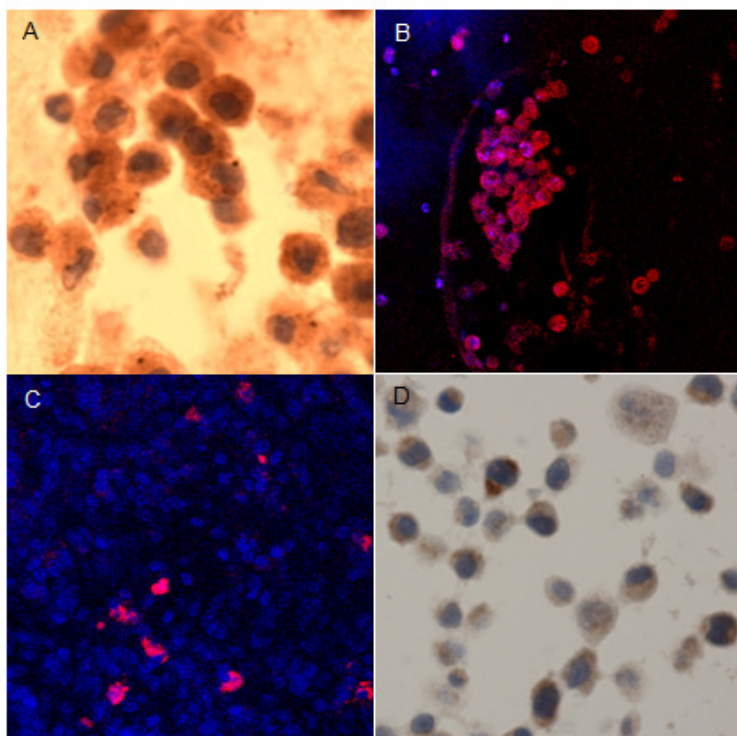

Supplementary Figure 3

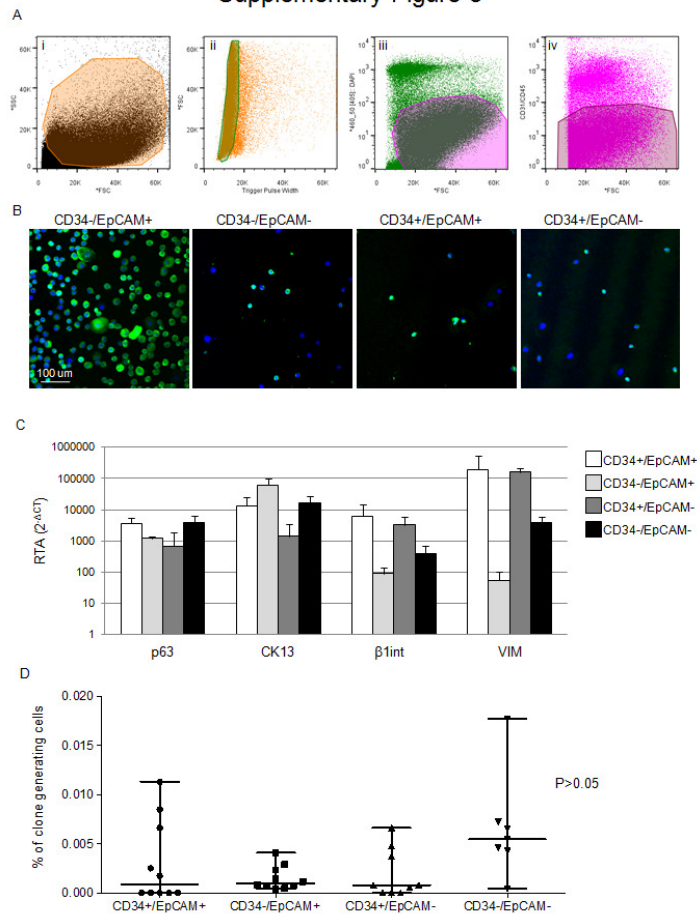

Supplement: Web figures [file gutjnl-2013-306171-s2.pdf]
