# Supplementary material for: The human squamous oesophagus has widespread capacity for clonal expansion from cells at diverse stages of differentiation
Source: Gut. 2014 Feb 26;64(1):11–9. doi: 10.1136/gutjnl-2013-306171 (PMC4283695; doi:10.1136/gutjnl-2013-306171)
Supplement: Web table 1 [file gutjnl-2013-306171-s3.pdf]

Supplementary table 1

Antigen	Species	Clonality	Conjugation	Manufacturer	Technique	Sample stained	Dilution
**Ki67**	Mouse	Monoclonal (MIB-1)	-	Dako	IF, IHC	Wholemounts<br>Primary cell clones<br>3D organotypic cultures	1:100
**Ki67**	Mouse	Monoclonal (SP6)	-	Abcam	IF	Wholemounts	1:250
**PH3**	Rabbit	Polyclonal	-	Millipore Upstate	IF	Wholemounts	1:250
**β1-integrin**	Mouse	Monoclonal (4B7R)	FITC	Santa Cruz	IF	Wholemounts	1:100
**MCSP**	Mouse	Monoclonal (9.2.27)	-	BD Bioscience	IF	Wholemounts	1:100
**PanCK**	Rabbit	Polyclonal	-	Acris Antibodies	IF, IHC	Wholemounts<br>Primary cell clones<br>3D organotypic cultures	1:500
**CD34**	Mouse	Monoclonal (581)	-	BD Bioscience	IF	Wholemounts	1:100
**CD31**	Mouse	Monoclonal (CJ70A)	-	Dako	IF	Wholemounts	1:200
**CD45**	Mouse	Monoclonal (ZB11+PD7/26)	-	Dako	IF	Wholemounts	1:100

**Chromogranin-A**	Rabbit	Monoclonal (DRM015)	-	Acris Antibodies	IF	Wholemounts	1:50
**F4/80**	Rat	Monoclonal (A3-1)	-	Abcam	IF	Wholemounts	1:200
**S-100**	Rabbit	Polyclonal	-	Abcam	IF	Wholemounts	1:100
**CK13**	Mouse	Monoclonal	-	Novocastra	IF, IHC	Primary cell clones 3D organotypic cultures	1:200
**CK8/18**	Mouse	Monoclonal (5D3)	-	Vector laboratories	IF	Primary cell clones	1:100
**Vimentin**	Rabbit	Monoclonal (VP-RM17)	-	Vector laboratories	IF, IHC	Primary cell clones 3D organotypic cultures	1:250
**EpCAM**	Mouse	Monoclonal (VU1D9)	-	Cell Signalling	IHC	Normal oesophagus	1:500
**β1-integrin**	Mouse	Monoclonal (18/CD29)	-	BD Bioscience	IHC	3D organotypic cultures	1:100
**p63**	Mouse	Monoclonal (4A4)	-	Dako	IHC	3D organotypic cultures	1:100
**MCM2**	Mouse	Monoclonal	-	Prof.s Steve Dilworth and Ron Laskey	IHC	3D organotypic cultures	1:500
**EpCAM**	Mouse	Monoclonal (9C4)	PE	Biolegend	FC	Fresh primary cells	1:25

**CD34**	Mouse	Monoclonal (581)	FITC	Biolegend	FC	Fresh primary cells	1:500
**CD31**	Mouse	Monoclonal (WM59)	Biotin	Biolegend	FC	Fresh primary cells	1:500
**CD45**	Mouse	Monoclonal (HI30)	Biotin	BD Bioscience	FC	Fresh primary cells	1:10
