# Supplementary material for: The human squamous oesophagus has widespread capacity for clonal expansion from cells at diverse stages of differentiation
Source: Gut. 2014 Feb 26;64(1):11–9. doi: 10.1136/gutjnl-2013-306171 (PMC4283695; doi:10.1136/gutjnl-2013-306171)
Supplement: Web table 2 [file gutjnl-2013-306171-s4.pdf]

**Supplementary table 2**

| Gene | Sequence | Annealing temp |
|---|---|---|
| **GaPDH F** | CAACAGCCTCAAGATCATCAG | 60° C |
| **GaPDH R** | ATGGACTGTGGTCATGAGTC | |
| **RPS18 F** | ATCCCTGAAAAGTTCCAGCA | 60° C |
| **RPS18 F** | CCCTCTTGGTGAGGTCAATG | |
| **β ACTIN F** | GGCATCCTCACCCTGAAGTA | 60° C |
| **β ACTIN R** | GGGGTGTTGAAGGTCTCAAA | |
| **CK13 F** | TGCAGATCGAGAGCCTGAATG | 60º C |
| **CK13 R** | TCCCTCATCTCTGCCAGCAC | |
| **β1 INTEGRIN F** | TGTGAATGCCAAAGCGAAGG | 60° C |
| **β1 INTEGRIN R** | CCAACACGCCCTTCATTGC | |
| **VIMENTIN F** | ACCGGAGACAGGTGCAGTCC | 60° C |
| **VIMENTIN R** | GCAGCTTCAACGGCAAAGTTC | |
| **P63 F** | GAGGTTGGGCTGTTCATCAT | 60° C |
| **P63 R** | AGGAGATGAGAAGGGGAGGA | |
